# State-of-the-Art Review: Native Joint Septic Arthritis Epidemiology, Pathogenesis, Microbiology, Diagnosis, Management, and Outcomes

**DOI:** 10.1093/cid/ciag473

**Published:** 2026-10-28

**Authors:** Ryan B Khodadadi, Takahiro Matsuo, Kaitlyn C Reasoner, Lauren Nicholas Herrera, Ceylan Colak, Katie D White, Brandon J Yuan, Aaron J Tande

**Affiliations:** Division of Infectious Diseases, Department of Medicine, Vanderbilt University Medical Center, Nashville, Tennessee, USA; Department of Infectious Diseases, Infection Control and Employee Health, The University of Texas MD Anderson Cancer Center, Houston, Texas, USA; Division of Infectious Diseases, Department of Medicine, Vanderbilt University Medical Center, Nashville, Tennessee, USA; Division of Infectious Diseases, Department of Medicine, Vanderbilt University Medical Center, Nashville, Tennessee, USA; Department of Radiology, Mayo Clinic, Rochester, Minnesota, USA; Division of Infectious Diseases, Department of Medicine, Vanderbilt University Medical Center, Nashville, Tennessee, USA; Department of Orthopedic Surgery, Mayo Clinic, Rochester, Minnesota, USA; Division of Public Health, Infectious Diseases, and Occupational Medicine, Mayo Clinic, Rochester, Minnesota, USA

**Keywords:** native joint septic arthritis, epidemiology, pathogenesis, microbiology, diagnostic evaluation, synovial fluid analysis, molecular diagnostics, source control, antimicrobial therapy, outcomes

## Abstract

Native joint septic arthritis (NJSA) is a time-sensitive orthopedic and infectious diseases emergency associated with substantial morbidity, mortality, and long-term functional impairment. This State-of-the-Art Review synthesizes current evidence on NJSA epidemiology with a primary focus on bacterial etiologies, risk factors, pathogenesis, microbiology, clinical presentation, and diagnostic challenges, including the absence of validated diagnostic criteria and limitations of culture-based evaluation. We summarize current diagnostic approaches with a focus on synovial fluid interpretation and the expanding role of molecular diagnostics. Management principles emphasize timely surgical source control, appropriate antimicrobial therapy, and coordinated multidisciplinary care. Outcomes are reviewed through the lens of relapse, reinfection, and functional recovery, highlighting the influence of host factors, joint involvement, and timeliness of intervention. Finally, we identify persistent gaps in evidence including optimal surgical timing, treatment duration, oral transition, clinical utility of emerging diagnostics, and outline research priorities to improve NJSA care and outcomes.

## Teaser

This State-of-the-Art Review synthesizes contemporary evidence on native joint septic arthritis epidemiology, microbiology, diagnosis, and management. We highlight diagnostic limitations, emerging molecular tools, surgical and antimicrobial strategies, outcomes, and research priorities needed to optimize timely care and improve functional recovery.

## Graphical Abstract

**Graphical Abstract:**
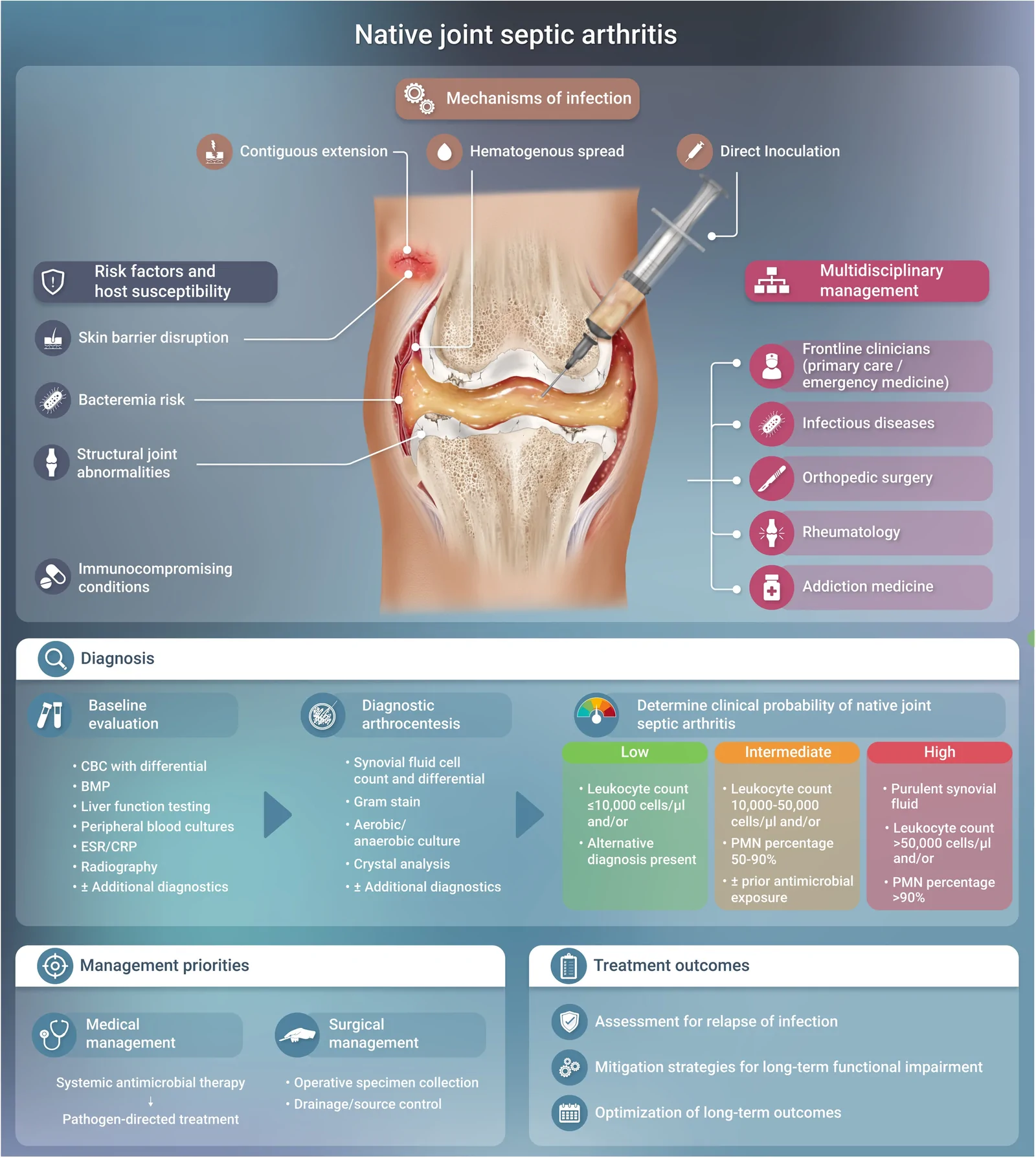

Native joint septic arthritis (NJSA) is a time-sensitive orthopedic and infectious diseases emergency characterized by rapidly progressive joint infection and substantial morbidity and mortality [1–3]. Without timely recognition, joint drainage, and antimicrobial therapy NJSA can result in irreversible joint destruction, long-term functional impairment, and death underscoring the need for prompt diagnosis and treatment [4–6].

Despite this clinical urgency, NJSA remains diagnostically challenging. Presentations often overlap with other causes of acute mono- or polyarthritis, and no universally accepted diagnostic criteria exist beyond isolation of a pathogenic organism [1, 3, 7, 8]. Although synovial fluid analysis frequently demonstrates abnormalities suggestive of infection, these findings lack specificity and may be difficult to interpret in isolation [3, 7–9]. Compounding these challenges, guideline-directed management in adults is limited and high-quality evidence informing key medical and surgical decisions remains sparse [8, 10–12].

In this State-of-the-Art Review, we synthesize current understanding of NJSA epidemiology, risk factors, pathogenesis, microbiology, clinical presentation, diagnostic evaluation, evidence-based management, and treatment outcomes in adults. We focus primarily on bacterial NJSA, highlighting special diagnostic considerations, and emphasize the importance of a collaborative, multidisciplinary approach required for timely and effective management of this potentially fatal condition. Lyme, mycobacterial, fungal, parasitic, and viral causes of NJSA as well as prosthetic joint infections and axial/spine infections are not specifically addressed in this State-of-the-Art Review as diagnostic and therapeutic approaches differ.

## EPIDEMIOLOGY AND BURDEN OF DISEASE

Exact epidemiologic estimates of NJSA are limited by its relative rarity, which constrains large prospective studies and results in reliance on retrospective analyses [3, 9, 13, 14]. Additional variability stems from heterogeneous case definitions, with many studies using Newman's modified criteria to include both culture-confirmed cases and clinically compatible presentations with purulent synovial fluid, including those following antimicrobial exposure or identified postmortem [1, 3].

Despite these limitations, population-based studies consistently characterize NJSA as uncommon, with an incidence of approximately 2–15 cases per 100 000 person-years [13, 15–18]. Incidence rises markedly in older adults (≥65 years) and in individuals with comorbidities or adverse social determinants of health [9]. More recently, increasing injection drug use (IDU) associated with the opioid epidemic has contributed to rising NJSA rates in some regions, with accompanying shifts in patient demographics and microbiology [19].

## RISK FACTORS AND HOST SUSCEPTIBILITY

Risk factors shape not only who develops NJSA but also how infection enters the joint, causative microbiology, and joints typically affected (Table 1). Although numerous NJSA risk factors have been described, they can be pragmatically grouped into 4 categories [3, 7, 9, 16, 33, 34, 37].

**Table 1.** Influence of Risk Factors, Epidemiologic Exposures, and Mechanisms of Infection on Microbiology and Joint Distribution of Native Joint Septic Arthritis

Risk Factor/Epidemiologic Exposure	Typical Mechanism Of Infection	Microbiologic Associations	Distinctive Joint Distribution/Pattern	Notes
Structural joint abnormalities (OA, RA, crystalline arthropathies, SLE, prior trauma/surgery)	Hematogenous	*Staphylococcus aureus*; β-hemolytic streptococci	Large joints (knee > hip/shoulder); ankle/wrist	Damaged synovium/cartilage predisposes to hematogenous seeding; RA adds risk via immunosuppression; RA disease flares may mimic NJSA [7, 20–24].
Injection drug use	Hematogenous (± embolic)	*Staphylococcus aureus* (MSSA/MRSA); Streptococci; water-borne Gram-negative organisms (historically *Pseudomonas aeruginosa,* more recently *Serratia* species)	Girdle (SC/AC/SI) and axial joints (facet); ↑ risk of oligo-/polyarticular involvement	High staphylococcal colonization rates; nonsterile injection practices promote transient bacteremia; consider endocarditis [19, 25–30].
Hemodialysis	Hematogenous	*Staphylococcus aureus* (MRSA); CoNS; Gram-negative organisms; line-associated pathogens	Large joints; ↑ risk of oligo-/polyarticular involvement	Access-related bacteremia → hematogenous seeding [31].
Ingestion of unpasteurized dairy products	Hematogenous	*Brucella* species	SI joint involvement	Exposure often travel-related; chronic/subacute course typical.
Sexually active individuals (suspected gonococcal arthritis)	Hematogenous	*Neisseria gonorrhoeae*	Migratory arthritis; flexor tenosynovitis; arthritis-dermatitis syndrome; small or large joints	Most common in younger adults; obtain NAAT at exposed GU and extragenital sites; culture yield low [32].
Exposure to *Ixodes* ticks in endemic regions (Northeast, upper Midwest), preceding erythema migrans rash	Hematogenous	*Borrelia burgdorferi*	Knee monoarthritis > other large joints	Two-tier serology for diagnosis; synovial PCR optional (lower sensitivity); antimicrobial-refractory arthritis possible (immune-mediated).
Diabetes mellitus	Hematogenous (±contiguous spread)	*Staphylococcus aureus*; streptococci; Gram-negative organisms (including *Pseudomonas aeruginosa*); polymicrobial infection	Large joints; small joints (MCP/IP/MTP/Ankle); ↑ risk of oligo-/polyarticular involvement	Impaired neutrophil function; vascular disease predisposes to bacteremia; atypical organisms possible [7, 33, 34].
Immunocompromising/immunomodulating conditions (CKD/ESRD, cirrhosis, HIV, malignancy, glucocorticoids/biologics)	Hematogenous (± contiguous spread)	Broader Gram-negative spectrum including GI/GU pathogens; atypical/opportunistic pathogens (fungi/mycobacteria)	Large joints; ↑ risk of oligo-/polyarticular involvement	Blunted inflammatory response; delayed diagnosis; risk for bacteremia GI/GU source; presence of intravascular access ↑ risk [7, 21, 33, 34].
Recent orthopedic surgery or intraarticular procedures	Direct inoculation	Staphylococci (including CoNS); streptococci; occasional polymicrobial	Joint accessed during procedure	Risk related to technique sterility; CoNS may represent true infection [17, 18, 21].
Traumatic arthrotomy, human bites, animal bites	Direct inoculation (±contiguous spread)	Polymicrobial; *Pasteurella*, *Capnocytophaga*, *Eikenella*; staphylococci/streptococci; anaerobes	Hand (MCP/IP); injured joint(s)	High mixed flora risk; may prove difficult to aspirate prior to surgery; early irrigation and debridement [21, 35, 36].
Skin barrier disruption: chronic exfoliative skin disorders (eg, psoriasis, eczema), ulcers	Contiguous spread	Polymicrobial; staphylococci/streptococci; anaerobes	Joint(s) adjacent to condition	Chronic skin breakdown allows for local extension [21].

Abbreviations: AC, acromioclavicular; CKD, chronic kidney disease; CoNS, coagulase-negative staphylococci; ESRD, end-stage renal disease; GI, gastrointestinal; GU, genitourinary; HIV, human immunodeficiency virus; IP, interphalangeal; MCP, metacarpophalangeal; MRSA, Methicillin-resistant *Staphylococcus aureus*; MSSA, Methicillin-susceptible *Staphylococcus aureus*; MTP, metatarsophalangeal; NAAT, nucleic acid amplification test; NJSA, native joint septic arthritis; OA, osteoarthritis; PCR, polymerase chain reaction; RA, rheumatoid arthritis; SC, sternoclavicular; SI, sacroiliac; SLE, systemic lupus erythematosus.

### Structural Joint Abnormalities

Structural joint abnormalities including osteoarthritis (OA), prior trauma/surgery, hip/knee prostheses, crystalline arthropathies, systemic lupus erythematosus, and rheumatoid arthritis (RA) are well-established risk factors for NJSA [7, 20, 21]. Preexisting synovitis, cartilage damage, and altered joint architecture facilitate hematogenous seeding during bacteremia [3, 22]. RA confers particularly high risk due to chronic joint damage, immunosuppressive therapies, and diagnostic overlap with disease flares [20, 21].

### Conditions Associated With Bacteremia

Conditions associated with bacteremia substantially increase NJSA risk. Persons who inject drugs (PWID) experience repeated transient or sustained bacteremia due to nonsterile injection practices [19, 25]. Similar risks are seen in patients with indwelling intravascular devices, including those receiving hemodialysis [31].

### Skin Barrier Disruption

Disruption of the skin barrier predisposes to NJSA via direct inoculation or contiguous spread of infection into the joint space including local trauma, recent joint injections or surgery, dermatologic disease including chronic exfoliative skin disorders (eg, psoriasis, eczema), and contaminated human or animal bites [21]. These infections more often involve the elbow and small joints of the hand (metacarpophalangeal [MCP] and interphalangeal joints [IP]) and are frequently polymicrobial [13].

### Immunocompromising Conditions

Immunocompromising conditions including advanced age, diabetes mellitus, chronic kidney disease, end-stage renal disease, cirrhosis, splenic dysfunction, alcohol use disorder, malignancy, human immunodeficiency virus, and iatrogenic immunosuppression including systemic corticosteroids, transplant-related immunosuppressive therapies, and biologic agents such as tumor necrosis factor-alpha inhibitors, broaden pathogen spectrum (notably Gram-negative bacilli and atypical organisms), increase bacteremia risk, and may attenuate signs and symptoms of infection, thereby delaying recognition and diagnosis of NJSA [7, 33, 34].

Despite these associations, approximately one-fifth of NJSA cases occur in patients without identifiable risk factors, underscoring the need for a high index of clinical suspicion in any adult with acute monoarthritis [37]. Understanding NJSA risk factors provides essential context for mechanisms by which pathogens enter the joint space.

## MECHANISMS OF INFECTION AND PATHOGENESIS

NJSA develops when pathogens gain access to the joint space via 1 of 3 primary mechanisms. Hematogenous seeding, the most common route, occurs when organisms enter the joint through the highly vascularized synovium, which lacks a basement membrane therefore permitting pathogen penetration [3, 9, 38, 39]. Large joints such as the knee, hip, and shoulder, characterized by rich vascular supply and prone to cumulative mechanical stress, are particularly susceptible to hematogenous seeding [3, 38].

Direct inoculation typically follows trauma, arthroscopy, intra-articular injections or aspirations, or other joint-invasive procedures [2, 16–18, 22]. Contiguous spread results from extension of adjacent infection including skin and soft-tissue infections, bursitis, or deeper musculoskeletal infection and is most clinically relevant in patients with poor skin integrity or deep-seated infection [22, 34, 38].

Regardless of mechanism, pathogen entry triggers a rapid and destructive inflammatory response. Neutrophil infiltration and cytokine-mediated processes produce proteolytic enzymes and reactive oxygen species that degrade cartilage and synovial structures, causing local tissue injury [3, 38]. Concurrently, accumulation of inflammatory exudate raises intra-articular pressure, impairing perfusion and nutrient delivery. Without timely drainage, irreversible cartilage damage may occur within 3 days [3]. These combined effects of direct microbial toxicity and host-mediated inflammation underscore the urgency of prompt recognition and intervention [3, 38].

## MICROBIOLOGY

Although virtually any microorganism can cause NJSA, the microbiologic profile reflects the interplay of host-specific risk factors, route of infection, and pertinent epidemiologic exposures (Table 1). In adults, monomicrobial, hematogenous large joint NJSA caused by Gram-positive cocci remains most common. *Staphylococcus aureus* is consistently the predominant pathogen, accounting for approximately half of cases in large contemporary cohorts (Table 2) [13, 19, 40, 41]. Methicillin-susceptible *S. aureus* is more frequently isolated than methicillin-resistant *S. aureus* (MRSA), although reported MRSA prevalence varies from minimal representation in some cohorts to nearly 20% of cases in United States series during the opioid era and substantial proportions of *S. aureus* isolates in parts of Asia [19, 26, 27].

**Table 2.** Microbiology of Native Joint Septic Arthritis in Adults From Large Retrospective Cohorts

Microbiologic Category^a^	Infections Attributable To Pathogen Or Pathogen Group^b,c,d,e^ Percentage (Range Across Studies)
*Staphylococcus aureus*	57.8 (52.8–59.4)
Methicillin-susceptible *Staphylococcus aureus* (MSSA)	44.2 (41.5–49.7)
Methicillin-resistant *Staphylococcus aureus* (MRSA)	13.7 (6.9–17.9)
*Staphylococcus lugdunensis*	0.4 (0–1.4)
Other coagulase-negative staphylococci	3.1 (1.2–8.3)
*Streptococcus pyogenes* (Group A)	4.7 (2.3–14.7)
*Streptococcus agalactiae* (Group B)	5.6 (3.5–8.8)
Other β-hemolytic streptococci (C/G)	2.7 (2.1–5.1)
*Streptococcus pneumoniae*	2.1 (0–4.6)
Other streptococcus species	6.1 (4.4–9.6)
Enterococci	1.8 (0.5–2.5)
*Cutibacterium acnes*	0.4 (0–1.6)
Aerobic Gram-negative bacteria	5.3 (3.7–11.7)
* Neisseria gonorrhoeae*	0.7 (0.2–1.3)
* Pasteurella* species	1.0 (0.8–2.1)
* Pseudomonas aeruginosa*	1.9 (1.3–2.3)
Other aerobic Gram-negative bacteria	1.7 (0–7.1)
Enterobacterales	6.4 (4.1–15.4)
Anaerobic organisms	2.2 (0.5–9.4)
Polymicrobial infections	10.1 (1.1–28.0)
Miscellaneous organisms^f^	3.9 (2.3–7.0)
Total Gram-positive organisms	83.1 (79.6–89.4)
Total Gram-negative organisms	11.9 (7.9–27.3)
Aerobic Gram-negative bacteria	5.3 (3.7–11.7)
Culture negative overall^g^	24.7 (19.7–33.0)

Data represent pooled frequencies of 2490 isolates across 4 cohort studies (Ross, McBride, Khodadadi, Dubost; total n = 2490). Values are presented as overall percentages with ranges reflecting the minimum and maximum percentages reported across individual studies.

^a^Organism rows reflect distributions among culture-positive cases; polymicrobial episodes contribute more than 1 organism.

^b^Derived from Ross et al, OFID 2020; multicenter, culture-positive, isolate-based cohort with a comparatively high MRSA burden in an urban tertiary setting [19].

^c^Derived from McBride et al, CID 2020; single-center, episode-based design that reports both culture-negative burden and a high polymicrobial fraction (28% of culture-positives) [13].

^d^Derived from Khodadadi et al, CID 2024; multicenter, joint-based cohort reporting both preoperative culture yield (215/321) and operative culture yield (188/321) as well as organism distributions [40].

^e^Derived from Dubost et al, JBJS 2014; single-center, culture-positive, isolate-based series showing little change over time in organism distribution and no significant MRSA increase [41].

^f^Miscellaneous organisms include fungal pathogens, *Mycobacterium tuberculosis*, nontuberculous mycobacteria, *Brucella*, and other rare organisms not represented in major taxonomic categories.

^g^The culture-negative proportion is derived from studies reporting this outcome (McBride and Khodadadi); other composite values reflect culture-positive cases.

*Streptococcus* species represent the second most common cause of NJSA, with β-hemolytic Streptococci predominating over non-β-hemolytic strains [13, 19, 40, 41]. Following widespread pneumococcal vaccination, *Streptococcus pneumoniae* accounts for a minority of cases, typically in older adults and immunocompromised hosts with concurrent bacteremia [9, 42].

Isolation of coagulase-negative staphylococci (CoNS) from native joints often raises concern for contamination. However, in appropriate clinical contexts, particularly following intra-articular procedures, CoNS are increasingly recognized as true pathogens [17, 18].

Gram-negative bacilli account for 10%–20% of NJSA cases in large cohorts and are encountered more often in older adults, immunocompromised hosts, and those with hematogenous seeding from urinary or intra-abdominal sources, with Enterobacterales predominating [13, 19, 40, 41]. *Salmonella* species are uncommon overall but occur disproportionately in patients with hemoglobinopathies while *Kingella kingae* is primarily a pediatric pathogen [11].

Among PWID, Gram-negative organisms such as *Pseudomonas aeruginosa* and *Serratia* species may be introduced through waterborne or environmental contamination of diluents, rinse water, or injection paraphernalia [19, 28]. However, recent studies consistently identify *S. aureus* and other skin flora as leading etiologies of NJSA in this population, likely reflecting high staphylococcal colonization rates combined with frequent nonsterile injections, promoting transient bacteremia [19, 28, 29].

*Neisseria gonorrhoeae* remains an important cause of NJSA in sexually active individuals, in particular young adults. Diagnosis is frequently established through nucleic acid amplification tests (NAATs) and other molecular methods from urogenital or extragenital sites given superior sensitivity compared with conventional culture [43, 44].

Polymicrobial NJSA typically follows direct inoculation or contiguous spread from penetrating trauma, contaminated wounds, or animal and human bites and often includes mixtures of staphylococci, streptococci, environmental and oral anaerobes, and organisms such as *Pasteurella*, *Capnocytophaga*, or *Eikenella* species [35, 36].

Culture-negative NJSA remains common, occurring in over 20% of cases due to prior antimicrobial exposure, fastidious pathogens, low-inoculum infection, inadequate sampling, or limitations of conventional culture methods [9, 13, 40]. When cultures are negative despite high clinical suspicion, further evaluation should be guided by epidemiologic exposures and host-specific risk factors and may include mycobacterial and fungal cultures or molecular diagnostic assays to investigate atypical etiologies of NJSA [32].

## CLINICAL PRESENTATION

Clinical manifestations of NJSA reflect the combined influence of host-specific risk factors, mechanism of infection and etiologic pathogens, which together shape disease tempo, symptomatology, and joint distribution (Table 1). Although NJSA occurs across clinical settings, it is most frequently encountered in emergency departments, where 8%–27% of patients with acute native joint pain are diagnosed with NJSA [45].

Symptom duration often correlates with underlying microbiology. Bacterial NJSA typically presents abruptly over hours to days, whereas subacute courses are more characteristic of *N. gonorrhoeae* and atypical pathogens including fungi, zoonoses, and mycobacteria [2, 3].

While several clinical features are sensitive for joint inflammation, their specificity is limited, as many noninfectious causes of acute mono- or polyarthritis may closely mimic NJSA including crystalline arthropathies (gout and calcium pyrophosphate deposition disease), RA flares, psoriatic arthritis, reactive arthritis, and hemarthrosis. These conditions often present with overlapping clinical features, including joint pain, swelling, and elevated inflammatory markers, which can complicate diagnostic assessment. A thorough clinical evaluation including detailed history, medication review, and careful physical examination with attention to skin findings is essential in distinguishing these entities. Importantly, the presence of a noninfectious diagnosis does not exclude concomitant infection. Localized joint pain and swelling are the most sensitive presenting features for NJSA and are often accompanied by erythema and warmth; fever, sweats, and rigors are variably present and insensitive, particularly among older adults and immunocompromised patients [7, 14, 22].

NJSA is usually monoarticular and involves large joints, most commonly the knee, followed by the hip, shoulder, wrist, and ankle [13]. Oligo- or polyarticular disease occurs in up to 20% of cases and is associated with worse outcomes, including septic shock, intensive care unit admission, and increased mortality [15, 23, 46]. In PWID, hematogenous seeding often involves girdle and axial joints (eg, sternoclavicular, acromioclavicular, sacroiliac) and may be oligo- or polyarticular, sometimes with concurrent endovascular infection or contiguous soft tissue infection at sites of injection [19, 25, 28, 29].

A comprehensive physical examination is essential. The most sensitive findings are pain with active and passive range of motion and joint effusion, which is readily appreciated in peripheral joints but often obscured in joints with substantial soft-tissue envelopes such as the hip and shoulder [7]. Erythema, warmth, axial-load pain, and focal tenderness are less sensitive and may be absent [45]. Evidence of a portal of entry or adjacent infection may suggest direct inoculation or contiguous spread.

## DIAGNOSTIC APPROACH AND CHALLENGES

### Baseline Evaluation

No single symptom or exam finding reliably confirms or excludes NJSA. When suspected, evaluation should prioritize prompt arthrocentesis with synovial fluid analysis alongside peripheral blood cultures and basic laboratory studies including complete blood count with differential, basic metabolic panel, liver function testing, and inflammatory markers such as C-reactive protein (CRP) and erythrocyte sedimentation rate to expedite diagnosis and guide early management.

### Synovial Fluid Analysis

Prompt arthrocentesis, ideally before antimicrobial administration is essential, with synovial fluid submitted for leukocyte count and differential [7, 24, 45]. These parameters support diagnosis but must be interpreted within clinical context, as no single leukocyte threshold is definitively diagnostic. Although higher synovial leukocyte counts increase the likelihood of infection, values below the traditional ≥50 000 cells/µL cutoff do not reliably exclude NJSA [7, 32, 45]. Recent studies suggest lower optimal thresholds of 27 000–33 000 cells/µL may be more appropriate [47, 48]. A polymorphonuclear (PMN) percentage of ≥ 90% is similarly supportive [45, 49]. Alternative metrics, including a synovial fluid neutrophil-to-lymphocyte ratio ≥25, may outperform conventional leukocyte count and PMN percentage thresholds for diagnosis [50].

Prior antimicrobial exposure may reduce synovial leukocyte counts, alter differentials, and decrease microbiologic yield [40, 51, 52]. Although no guideline-directed adjustments exist, lower leukocyte count thresholds (≥16 000 cells/µL) may improve sensitivity in this setting [47]. In a retrospective study of hip and knee NJSA, prior antimicrobial exposure reduced synovial leukocyte counts, but median PMN percentages remained elevated, supporting PMN predominance as a reliable marker despite antimicrobials [53].

In this framework, synovial fluid parameters are used to classify the clinical probability of NJSA: high probability findings include purulent synovial fluid, leukocyte counts >50 000 cells/µL, and/or polymorphonuclear (PMN) percentage ≥90%; intermediate probability includes leukocyte counts approximately 10 000–50 000 cells/µL and/or PMN percentage 50%–90%, including in the setting of prior antimicrobial exposure; and low probability includes leukocyte counts <10 000 cells/µL and/or the presence of a clear alternative diagnosis.

Patients with intermediate probability synovial fluid findings represent a diagnostic gray zone in which neither infection nor noninfectious inflammation can be reliably excluded. Specific leukocyte counts and PMN percentages should be evaluated on a continuum, with the post-test probability of NJSA adjusted accordingly. In this setting, clinical judgment should guide management, with prioritization of adjunctive diagnostic studies including Gram stain, culture, and blood cultures, as well as consideration of molecular diagnostics when available. Additional factors including host risk factors, presence of crystals, and systemic features of infection, should be integrated into decision-making.

Synovial glucose and protein do not reliably distinguish NJSA from noninfectious arthritis and should not be routinely ordered [7, 45, 49, 54]. Evidence supporting synovial lactate dehydrogenase is limited by low sensitivity [7, 49, 55]. Although synovial lactate ≥10 mmol/L has been proposed as suggestive of infection, interpretation depends on assay methodology and isoform reporting, and diagnostic performance is variable with elevations seen in other inflammatory conditions [24, 56, 57].

Routine synovial fluid studies should also include crystal analysis although the presence of crystals does not rule out NJSA [49]. Concomitant crystalline arthritis and NJSA is well described, with retrospective series reporting NJSA in >5% of aspirates positive for crystals [58–60]. Importantly, crystalline and other inflammatory arthropathies can mimic NJSA clinically and diagnostically, causing elevation of serum and synovial inflammatory markers, complicating diagnostic interpretation based on leukocyte counts alone [54, 61]. There is not an established synovial leukocyte count threshold to diagnose NJSA in the presence of concomitant crystals. In this setting, synovial leukocyte counts should be interpreted cautiously within the broader clinical context rather than relying on a single diagnostic threshold. Some studies support maintaining the traditional cutoff of ≥50 000 cells/µL threshold whereas other data suggests a higher threshold (≥85 000 cells/µL) to improve specificity [60, 61].

Gram stain and culture should always be performed on synovial fluid ideally with inoculation of fluid into aerobic and anaerobic blood culture bottles to improve culture yield [32]. Gram stain is highly specific and sensitivity is variable, as low as 26% in some series [62, 63]. Both Gram stain sensitivity and culture positivity can also be reduced by prior antimicrobial exposure [52, 62]. Culture sensitivity has been reported as high as 95%, although this is variable based on causative organism and antimicrobial exposure [9, 55].

Patients with suspected NJSA in whom arthrocentesis yields no synovial fluid for analysis should continue to be evaluated for NJSA, as a “dry tap” does not exclude the diagnosis. Reasons for failed synovial fluid aspiration may include inaccurate needle placement, small volume of effusion, fluid viscosity, and/or synovial proliferation in patients with prolonged illness. Repeat image-guided arthrocentesis should be considered if the unsuccessful arthrocentesis was either performed without image guidance or needle placement was possibly inaccurate. Saline lavage has been used in an attempt to obtain fluid for analysis, although some guidelines specifically recommend against this practice [8]. Patients with high suspicion for NJSA in whom repeat arthrocentesis is unlikely to be successful should proceed to surgery.

Although synovial fluid studies from arthrocentesis typically guide initial diagnosis and inform surgical decision making, multiple intraoperative tissue specimens should be collected and submitted for culture to aid in confirming or clarifying the diagnosis during surgery [64]. Swabs should not be used for specimen collection due to inferior diagnostic sensitivity [32]. When clinical suspicion for NJSA remains intermediate to high despite intermediate or nondiagnostic synovial fluid findings, early orthopedic evaluation and consideration of operative management should not be delayed.

### Additional Diagnostic Considerations

Peripheral blood cultures should be obtained in all suspected cases as concomitant bacteremia is common and, in some cases, may identify the pathogen [9, 37]. Echocardiography may be most helpful in patients with sepsis, shock, and positive blood cultures (particularly *S. aureus)*, persistent bacteremia, cardiac implantable electronic devices or prosthetic heart valves where the pretest probability of endovascular infection is higher [30].

*N. gonorrhoeae* is difficult to recover by conventional culture, purported to be due to the fastidious nature of the organism, although immunologically mediated arthritis is also a possibility [9, 22, 43, 44]. When suspected, NAAT should be performed on synovial fluid if available and at exposed urogenital and extragenital sites [32]. *N. gonorrhoeae* is also included on a recently Food and Drug Administration (FDA)–cleared multiplex polymerase chain reaction (PCR) panel [32].

Less common etiologies of NJSA including *Mycobacterium tuberculosis*, *Brucella* species, *Tropheryma whipplei*, and *Borrelia burgdorferi* may require additional diagnostic testing and pathogen-specific testing should be guided by the combination of epidemiologic exposures, host-specific risk factors, and compatible clinical presentation [2, 9, 32].

### Advances in Diagnostics

Given the frequency of culture-negative NJSA, molecular diagnostics can serve as valuable supplementary testing when routine Gram stain and cultures are unrevealing. In such cases, multiplex PCR panel testing or bacterial 16S ribosomal ribonucleic acid PCR and sequencing from orthopedic specimens can be considered and are readily available in some clinical environments [32]. The addition of next generation sequencing can increase pathogen detection as compared with Sanger sequencing alone and may be more sensitive than culture [65].

A recently FDA-approved multiplex PCR panel is commercially available in the United States and Europe and has demonstrated high sensitivity and specificity in multiple studies [66–68]. A commonly cited limitation of this panel is the omission of *Staphylococcus epidermidis* and *Cutibacterium acnes*, organisms more frequently implicated in prosthetic joint infection [66–68]. Common culprit pathogens of NJSA in pediatric patients such as *K. kingae* and *Haemophilus influenzae* are included on this panel and positive results in adults require cautious interpretation [66–68]. Overall, advanced molecular testing is not intended to replace or negate the need for standard cultures but can be useful in challenging cases with fastidious pathogens or in patients who may have limited culture yield due to prior antimicrobial exposure [65].

Shotgun metagenomic sequencing (sMGS) of plasma microbial cell-free deoxyribonucleic acid (mcfDNA) in peripheral blood is an emerging diagnostic strategy in musculoskeletal infection, including NJSA [69, 70]. Although real-world data remain limited, sMGS of mcfDNA may increase diagnostic yield in NJSA and other osteoarticular infections but requires further validation [69]. In a recent study, synovial fluid metagenomic next-generation sequencing outperformed culture (65.9% vs 33.3%), particularly at leukocyte counts <25 000/µL and in culture-negative cases, however, it remains an adjunct as cultures remain essential for antimicrobial susceptibility testing [70]. Additional research and clinical experience will be necessary to further establish the role of advanced molecular testing in NJSA diagnosis.

### Imaging

Imaging plays an important adjunctive role in NJSA diagnosis, defining disease extent, and guiding aspiration [71]. Although findings may overlap with noninfectious conditions such as inflammatory arthritis or postsurgical change, imaging helps confirm effusion, identify complications, and facilitate sampling and may also define predisposing risk factors such as penetrating trauma or adjacent soft tissue infection/abscess [71].

Radiography is the recommended initial modality and should always be obtained when NJSA is suspected. Early films are often unremarkable and insensitive for intra-articular infection, but radiographs may demonstrate effusion in superficial joints and later findings such as periarticular osteopenia, joint-space narrowing from cartilage loss, and marginal erosions (Figure 1*A*) [71]. In selected cases with supportive findings, radiography may provide sufficient diagnostic information to guide management without the need for additional advanced imaging studies.

**Figure 1.**
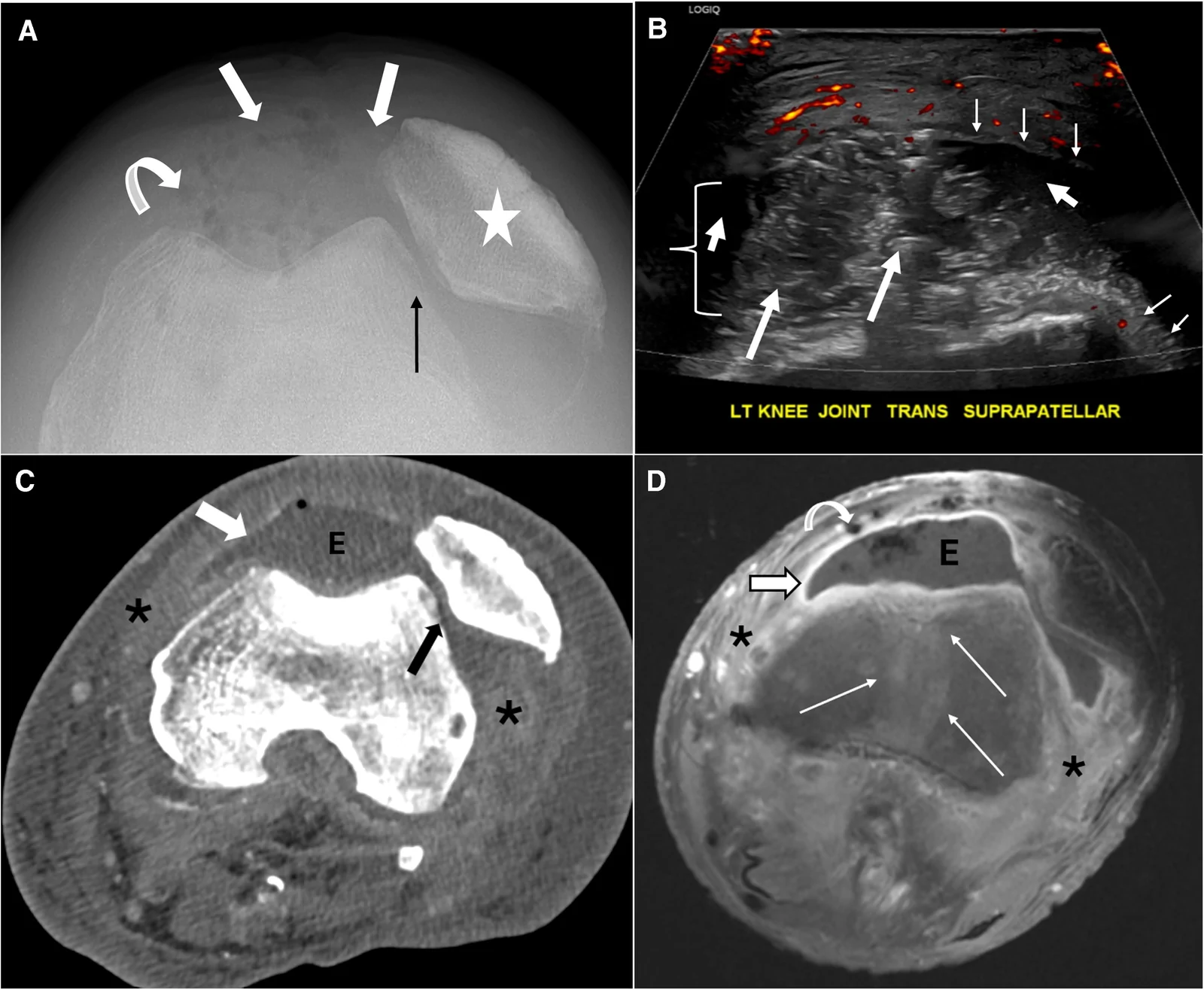
Multimodal imaging findings in native joint septic arthritis. Abbreviations: CT, computed tomography; MRI, magnetic resonance imaging. *A*, Radiograph (sunrise view): shows a large suprapatellar joint effusion (white arrows), subtle marginal bone erosions along the patellofemoral compartment (thin black arrow) and associated lateral patellar subluxation (star). Air bubbles following knee aspiration are also visible (curved arrow). *B*, Ultrasound: demonstrates a complex joint effusion with internal debris (long thick arrows), synovial thickening (short thin arrows), joint fluid (short thick arrows), and marked synovial hyperemia on Power Doppler, consistent with active synovitis. The brace denotes fluid mixed with debris. There is increased red Doppler signal in the adjacent soft tissue due to inflammation. *C*, Contrast-enhanced axial CT: reveals a large effusion (E), enhancing and thickened synovium (white arrow), and adjacent ill-defined soft-tissue edema and enhancement (asterisk). Tiny erosive changes are visible along the lateral femoral cortex (black arrow). *D*, Postcontrast axial MRI: shows a large joint effusion (E) with susceptibility artifact from intra-articular air introduced during prior aspiration (curved arrow). Additional findings include synovial thickening and enhancement (short thick arrow), surrounding soft-tissue edema (asterisk), and pronounced bone marrow edema (long thin arrows) with enhancement, consistent with reactive osteitis or early osteomyelitis.

Ultrasound is highly useful for detecting joint effusions and has a high negative predictive value when no effusion is present [72]. Power Doppler may demonstrate synovial hyperemia, though this is nonspecific (Figure 1*B*) [72]. Ultrasound also enables real-time, low-risk image-guided arthrocentesis while fluoroscopic or computed tomography (CT) guidance may be required for deeper or anatomically complex joints such as sternoclavicular or sacroiliac joints [71]. CT, while not routinely requested, may also reveal bony erosions, intra-articular gas, or adjacent fluid collections (Figure 1*C*).

Magnetic resonance imaging (MRI) is the most sensitive modality for early infection, particularly for deeper or anatomically complex joints such as axial joints, demonstrating joint effusion, synovial thickening and enhancement, subchondral marrow edema, and perisynovial soft-tissue involvement (Figure 1*D*, Supplementary Figure 1) [71, 73]. Although specificity is limited, contrast-enhanced MRI is preferred when differentiating infection from sterile inflammatory arthropathies or suspected complications such as osteomyelitis [73].

Nuclear medicine studies including three-phase bone scan, gallium-67 scintigraphy, labeled leukocyte imaging, single-photon emission computed tomography/computed tomography, and fluorine-18 fluorodeoxyglucose positron emission tomography/computed tomography (FDG PET/CT) may assist in challenging or postoperative cases, multifocal disease, or treatment response, though specificity remains limited [71, 74, 75].

## MEDICAL MANAGEMENT

A diagnostic and management algorithm for NJSA is presented in Figure 2, summarizing key decision points from initial evaluation through antimicrobial and surgical management. Empiric antimicrobial therapy, defined as antimicrobial treatment once the diagnosis is suspected but not yet fully defined, should be administered in all patients ideally after blood and synovial fluid samples have been sent for culture [8]. Early empiric antimicrobial therapy is supported by the high frequency of concomitant bloodstream infection (40%–50%) and animal model data suggesting progressive joint destruction with treatment delay [40, 76]. As with all empiric antimicrobial therapy, the goal is to maximize causative pathogen coverage while minimizing unnecessary antimicrobial exposure.

**Figure 2.**
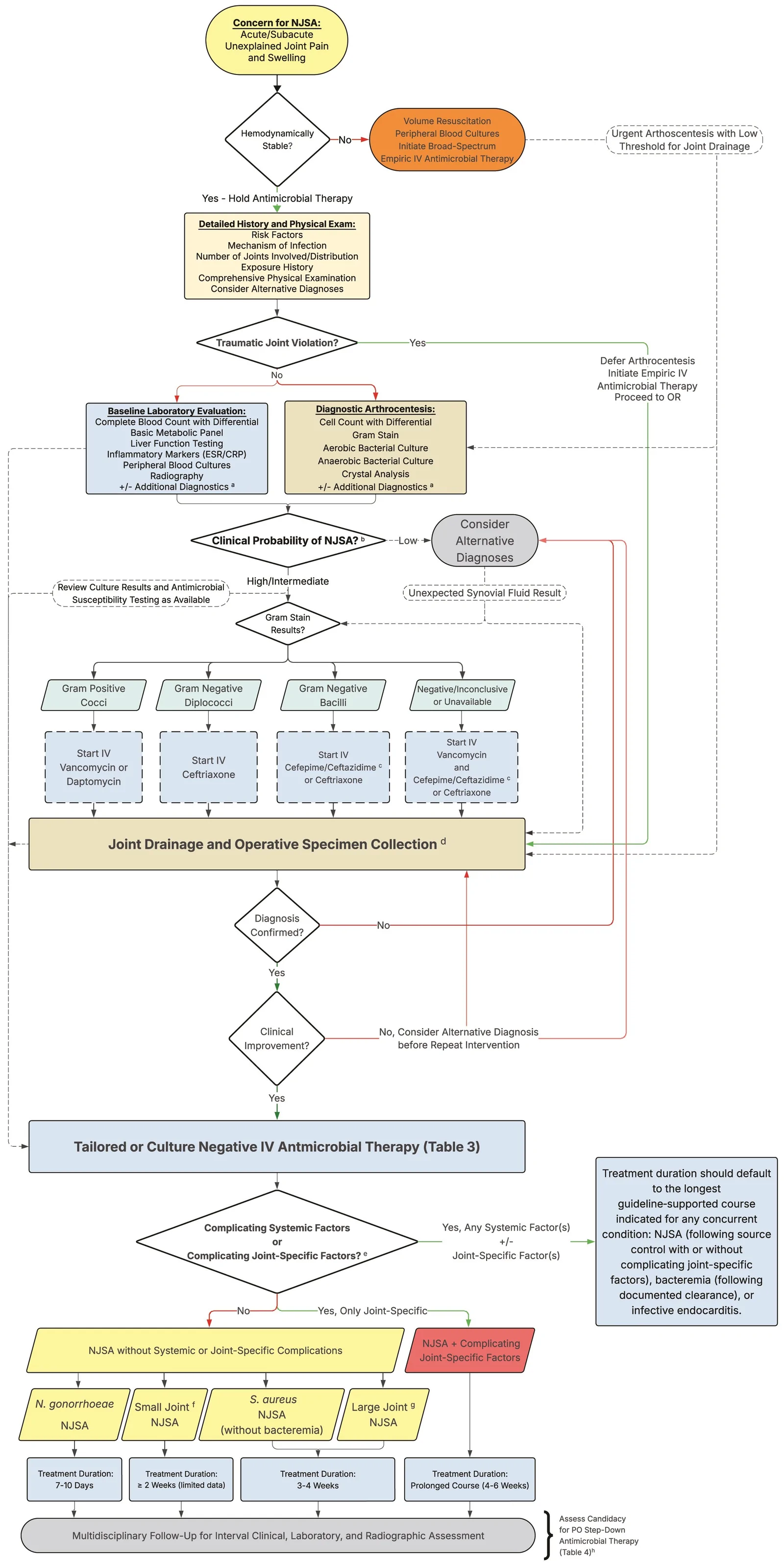
Proposed diagnostic and management algorithm for suspected native joint septic arthritis. Abbreviations: NJSA, native joint septic arthritis; IV, intravenous; OR, operating room; ESR, erythrocyte sedimentation rate; CRP, C-reactive protein; cells/µL, cells per microliter; *S. aureus*, *Staphylococcus aureus*; *N. gonorrhoeae, Neisseria gonorrhoeae;* PO, per os. ^a^Additional diagnostics may include fungal culture, mycobacterial culture, and molecular assays depending on pertinent epidemiological exposures. ^b^Synovial fluid parameters are used to classify the clinical probability of NJSA: high-probability findings include purulent synovial fluid, leukocyte counts >50,000 cells/μL, and/or a polymorphonuclear (PMN) percentage ≥90%; intermediate-probability findings include leukocyte counts of approximately 10,000 to 50,000 cells/μL and/or a PMN percentage of 50% to 90%, including in the setting of prior antimicrobial exposure; and low-probability findings include leukocyte counts <10,000 cells/μL and/or the presence of a clear alternative diagnosis. The intermediate leukocyte count range reflects the heterogeneity of thresholds reported across published studies. ^c^The choice between antipseudomonal (cefepime or ceftazidime) and non-antipseudomonal (ceftriaxone) third- or fourth-generation cephalosporins should be guided by individual risk factors for *Pseudomonas* infection including: a prior positive *Pseudomonas* culture in the past 12 months (any site), immunosuppression, persons who inject drugs, severe burns, sepsis or septic shock, recent hospitalization and IV antimicrobial exposure within the previous 90 days. ^d^Operative specimens should be submitted for Gram stain, aerobic culture, anaerobic culture, and, when clinically indicated, fungal culture, mycobacterial culture, and molecular diagnostics. ^e^Complicating systemic factors include concomitant bacteremia, *S. aureus* NJSA with bacteremia, endovascular infection, and multiple foci of infection. Complicating joint-specific factors include periarticular/contiguous osteomyelitis, difficult-to-treat pathogens, and multiple operative interventions. ^f^Small joints refer to joints such as the metacarpophalangeal (MCP), proximal interphalangeal (PIP), distal interphalangeal (DIP), and metatarsophalangeal (MTP) joints. ^g^Large joints refer to joints such as the hip, knee, shoulder, and ankle. ^h^Transition from IV to PO antimicrobial therapy may be considered once patients demonstrate clinical stability with improving signs and symptoms of infection, have an available active PO agent supported by susceptibility data, and lack significant allergies, intolerances, or drug–drug interactions. Patients should be able to reliably tolerate PO medications without impaired absorption, and infection should be adequately source controlled with clearance of bloodstream infection in gram-positive cases. Transition is individualized but is often reasonable after approximately 7–10 days of initial IV therapy, guided by patient- and infection-specific factors.

Synovial fluid Gram stain is rapidly available in most clinical settings and can help direct empiric antimicrobial therapy. Patients with Gram-positive cocci on Gram stain should receive vancomycin or daptomycin to avoid MRSA treatment delays [77]. Immunocompetent patients without trauma or IDU in whom Gram-negative bacilli are identified may receive ceftriaxone, whereas those with these risk factors should receive cefepime or ceftazidime. Colonization with resistant Gram-negative bacilli should also inform empiric selection. Patients with a negative Gram stain should receive vancomycin or daptomycin plus a third or fourth generation cephalosporin, selected based on the aforementioned risk factors.

Unlike large joint NJSA, infections of small joints of the hands and feet are more frequently polymicrobial, including anaerobic pathogens [13]. Empiric antimicrobial therapy should therefore be guided by the associated infectious syndrome, such as diabetic foot infection, traumatic upper-extremity injuries or animal bites. For example, ampicillin-sulbactam monotherapy would be appropriate empiric antimicrobial therapy for MCP NJSA associated with a dog or cat bite.

Transitioning from empiric to definitive antimicrobial therapy depends on peripheral blood, preoperative synovial fluid, and operative culture results. Antimicrobial therapy can often be narrowed to a single agent among patients with positive blood cultures for a known pathogen and presumed hematogenous infection. When available and performed, rapid multiplex PCR testing may facilitate early de-escalation; for example, detection of *S. aureus* with negative *mecA* testing permits early selection of definitive therapy. In most cases, however, definitive antimicrobial therapy requires pathogen identification and antimicrobial susceptibility testing.

Initial intravenous therapy is typical while awaiting clinical improvement. However, subsequent oral therapy is effective and noninferior when suitable agents are available and no other indication for intravenous therapy exists [78, 79]. Transition to oral therapy after 7–10 days is reasonable, extrapolating from the Oral Versus Intravenous Antibiotics (OVIVA) trial [79]. However, in many cases, while the OVIVA trial supports the safety and efficacy of oral step-down therapy, the optimal timing and transition is often individualized. Factors influencing this transition include clinical stability, resolution of fever, ability to tolerate oral medications, availability of active agents with favorable oral bioavailability, source control, and microbiologic data [80]. Accordingly, a uniform approach may not adequately account for clinical variability, and decisions regarding route and duration of therapy should be guided by patient- and infection-specific factors (Figure 2) [80]. Tables 3 and 4 summarize intravenous and oral options for definitive antimicrobial therapy for selected pathogens.

**Table 3.** Intravenous Antimicrobials Used for Treatment of Native Joint Septic Arthritis

Antimicrobial	Recommended Dose^a^	Targeted Organisms
Ampicillin	12 g over 24 h in continuous infusion or divided every 4–6 h	Susceptible streptococci and enterococci
Ampicillin-sulbactam	3 g every 6 h	MSSA; *Pasteurella*; *Capnocytophaga*; *Fusobacterium*; Streptococci; polymicrobial infections including anaerobes
Cefazolin	2 g every 8 h	MSSA; methicillin-susceptible CoNS; penicillin-susceptible streptococci
Cefepime	2 g every 8–12 h	*Pseudomonas aeruginosa*
Ceftriaxone	2 g every 24 h	Streptococci; Cutibacteria; susceptible Enterobacterales; some clinicians use for nonbacteremic MSSA
Ceftazidime	2 g every 8 h	*Pseudomonas aeruginosa*
Daptomycin	6–10 mg/kg every 24 h^b^	MRSA; enterococci (including VRE)
Ertapenem	1 g every 24 h	Enterobacterales, including ESBL strains; polymicrobial infections including anaerobes
Imipenem	500 mg every 6 h	*Pseudomonas aeruginosa;* ESBL-producing Enterobacterales; polymicrobial infections including anaerobes
Meropenem	1 g every 8 h	*Pseudomonas aeruginosa;* ESBL-producing Enterobacterales; polymicrobial infections including anaerobes
Nafcillin	1.5–2 g every 4–6 h	MSSA; methicillin-susceptible CoNS
Oxacillin	1.5–2 g every 4–6 h	MSSA; methicillin-susceptible CoNS
Penicillin G	20 million units over 24 h in continuous infusion or divided every 4 h	Penicillin-susceptible streptococci and enterococci; Cutibacteria
Piperacillin-tazobactam	3.375–4.5 g every 6 h^c^	*Pseudomonas aeruginosa;* Enterobacterales; polymicrobial infections including anaerobes
Vancomycin	15 mg/kg every 12 h^d^	MRSA; methicillin-resistant CoNS; also second-line for MSSA, methicillin-susceptible CoNS, streptococci and enterococci, Cutibacteria

Adapted with permission from Tande AJ, Suh GA, Osmon DR, Berbari EF, Steckelberg JM. Osteomyelitis in: Mandell, Douglas, and Bennett's Principles and Practice of Infectious Diseases. Netherlands: Elsevier Health Sciences. 2026. Chapter 106.

Antimicrobial selection should be based on many factors, including in vitro sensitivity, allergies and intolerances, drug interactions, adverse event risk, renal and hepatic function, and cost. The antimicrobial options listed are not exhaustive, and therapy should be individualized based on suspected or confirmed pathogens and clinical context.

Abbreviations: CoNS, coagulase-negative staphylococci; ESBL, extended-spectrum beta-lactamase; g, gram(s); h, hours; mg/kg, milligrams/kilogram; MRSA, methicillin-resistant *Staphylococcus aureus*; MSSA, methicillin-susceptible *Staphylococcus aureus*; VRE, vancomycin-resistant enterococcus.

^a^Doses provided for normal renal function; adjustment may be needed with reduced renal function.

^b^Higher doses recommended for MRSA and enterococci.

^c^Higher doses recommended for *Pseudomonas aeruginosa*.

^d^Dosing to be adjusted for renal function and therapeutic drug monitoring.

**Table 4.** Oral Antimicrobials Used for Treatment of Native Joint Septic Arthritis

Antimicrobial	Recommended Dose^a^	Targeted Organisms
Amoxicillin	1000 mg three times daily	Penicillin-susceptible streptococci and enterococci
Cefadroxil	1000 mg twice daily	MSSA; methicillin-susceptible CoNS; penicillin-susceptible streptococci
Ciprofloxacin	500–750 mg twice daily^b^	Enterobacterales, *Pseudomonas aeruginosa*
Clindamycin	600 mg three times daily	MSSA; CoNS
Doxycycline	100 mg twice daily	MSSA; CoNS
Levofloxacin	750 mg daily	Enterobacterales; *Pseudomonas aeruginosa; S. aureus* (only in combination with rifampin for susceptible isolates; not recommended as monotherapy)^c^
Linezolid	600 mg twice daily	Enterococci; MRSA
Minocycline	100 mg twice daily	MSSA; CoNS
Trimethoprim-sulfamethoxazole	8–10 mg/kg (of TMP component) in 2–3 divided doses daily	MSSA; CoNS; Enterobacterales

Adapted with permission from Tande AJ, Suh GA, Osmon DR, Berbari EF, Steckelberg JM. Osteomyelitis in: Mandell, Douglas, and Bennett's Principles and Practice of Infectious Diseases. Netherlands: Elsevier Health Sciences. 2026. Chapter 106.

Antimicrobial selection should be based on many factors, including in vitro sensitivity, allergies and intolerances, drug interactions, adverse event risk, renal and hepatic function, and cost. The antimicrobial options listed are not exhaustive, and therapy should be individualized based on suspected or confirmed pathogens and clinical context.

Abbreviations: CoNS, coagulase-negative staphylococci; mg, milligrams; MRSA, methicillin-resistant *Staphylococcus aureus*; MSSA, methicillin-susceptible *Staphylococcus aureus*; TMP, trimethoprim.

^a^Doses provided for normal renal function; adjustment may be needed with reduced renal function.

^b^Higher doses recommended for *Pseudomonas aeruginosa*.

^c^For susceptible *S. aureus* isolates, combination therapy with a fluoroquinolone and rifampin has been commonly used for staphylococcal infections in studies evaluating oral regimens, including in patients without orthopedic implants. The most frequently reported regimen is levofloxacin in combination with rifampin, and *in vitro* data supports the use of this combination. When rifampin cannot be used due to resistance or intolerance, alternative agents listed in the table for staphylococcal infection (excluding levofloxacin) may be administered as monotherapy, although supporting evidence for this approach is more limited. The optimal dosing strategy for rifampin remains uncertain; however, twice-daily dosing totaling 600–900 mg/d is commonly employed. Careful attention should be given to potential drug–drug interactions associated with rifampin, including those involving other antimicrobial agents [81–83].

Approximately 20% of NJSA cases lack an identified pathogen despite appropriate sampling, often due to antecedent antimicrobial exposure [13]. In such cases, data surrounding oral antimicrobial use is scarce and the optimal route of therapy remains uncertain. All prior antimicrobials should be considered when selecting subsequent definitive antimicrobial therapy. Decisions regarding intravenous versus oral therapy in culture-negative NJSA should be individualized and may reasonably incorporate a patient-centered approach, including shared decision-making that balances patient- and infection-specific factors. Less commonly, pathogens requiring specialized testing such as mycobacteria, fungi, or *Brucella* species, may result in negative cultures. In these patients, negative cultures and subacute clinical presentation should prompt epidemiological exposure assessment and adjunctive testing.

Following adequate joint drainage or debridement, optimal duration of antimicrobial therapy remains uncertain. Evidence-based guideline panels suggest a total treatment duration of 4–6 weeks, consistent with contemporary observational data [6, 8, 10, 13]. For most patients with large joint NJSA, 3–4 weeks of antimicrobial therapy is appropriate as shorter courses are associated with a higher rate of treatment failure [13, 84]. Data supporting shorter treatment durations are limited but suggest that, in selected patients with small joint NJSA, particularly involving the hand, 2 weeks of antimicrobial therapy following adequate surgical management may be sufficient [85, 86]. NJSA due to *N. gonorrhoeae* may be treated with 7–10 days of therapy, with the shorter duration of therapy supported by the rapidity of synovial fluid sterilization [87].

## SURGICAL MANAGEMENT

### Surgical Approaches and Indications

Surgical intervention is central to NJSA management, serving as the primary means of source control, reducing intra-articular inflammatory burden, and preventing irreversible cartilage loss. Historically, selected early uncomplicated cases were managed with serial needle aspiration; however, limited ability to clear loculated purulence and concerns for progressive chondrocyte injury led to broader adoption of operative drainage [3].

Open arthrotomy offers reliable exposure, evacuation, and synovectomy, but at the cost of greater soft-tissue disruption and postoperative stiffness. Contemporary practice has shifted toward arthroscopic irrigation and debridement for many large joints as it enables thorough lavage and synovectomy with less morbidity and earlier mobilization. In the knee, comparative data demonstrate infection control comparable to open arthrotomy, with advantages in early recovery [88, 89]. Hip arthroscopy can also be effective in experienced centers, though technical complexity and resource requirements are greater [89, 90]. Open arthrotomy remains preferable when dense adhesions, chronic or loculated infection, extra-articular extension, or concomitant osteomyelitis limit arthroscopic access [3, 88, 89].

Serial aspiration should be reserved for narrowly defined scenarios, such as very early (<48 hours) small-joint infection or when operative risk is prohibitive and requires close monitoring with a low threshold for operative conversion. Retrospective comparisons suggest no major outcome differences compared with surgery, though selection bias limits interpretation [91]. Technique selection should integrate joint anatomy, symptom duration, anatomic access, surgeon expertise, and presence of complex or loculated infection.

### Timing of Intervention

Experimental models demonstrate rapid early chondrocyte injury, supporting timely operative drainage [92]. Although some studies have not demonstrated a clear benefit of urgent surgery, other studies and expert guidance support prompt intervention, with delays beyond 48 hours potentially associated with worse functional and clinical outcomes [6, 8, 93–95]. In practice, surgical debridement should ideally be performed within 24 hours of diagnosis when feasible and when patient stability permits [6, 8].

### Management of Persistent Infection and Salvage Options

Despite appropriate initial treatment, 10%–38% of patients require additional surgery [96, 97]. Indicators of persistent infection include recurrent or persistent effusion, ongoing fever or bacteremia, and secondary rises in inflammatory markers. Risks for failure after a single debridement include large joint involvement, sepsis, synovial leukocyte count >85 000/µL, *S. aureus* infection, and diabetes [96, 97]. Repeat arthroscopy is reasonable for persistent knee or shoulder infection, but conversion to open arthrotomy is often necessary for inadequate arthroscopic debridement or loculated disease [96, 97].

When extensive cartilage destruction, severe degenerative disease, or chronic infection precludes joint preservation, salvage strategies may be required. Options include resection arthroplasty, arthrodesis, or staged total joint arthroplasty with an antimicrobial spacer; although the evidence base is limited, series report high eradication rates [98, 99].

## TREATMENT OUTCOMES

Treatment outcomes in NJSA vary widely due to differences in host-specific risk factors, microbiology, and timeliness of management. Although survival has improved substantially over recent decades, residual pain, functional deterioration, and relapse remain common [13]. Early recognition and coordinated medical and surgical therapy are central to preserving joint function and preventing disability.

### Definitions and Measurements of Treatment Success

Treatment failure in adult NJSA is variably defined across studies but generally refers to the persistence or recurrence of infection despite appropriate surgical and antimicrobial therapy. A recent international Delphi consensus defines failure as persistent physical signs of arthritis (eg, pain and swelling) and/or systemic inflammation (eg, fever and no improvement in CRP) despite surgical and antimicrobial treatment [100]. However, operational definitions differ across studies: some restrict failure to persistent infection during initial treatment course whereas others include relapse (recurrence), recurred infection in the same joint, typically with the same organism after clinical resolution, and reinfection, a new episode in the previously affected joint often caused by a different pathogen [13, 84, 85, 96]. Consequently, reported failure rates and outcome comparisons vary considerably, highlighting the need for standardized composite endpoints that integrate microbiologic clearance and functional recovery.

### General Prognosis and Determinants of Outcome

Outcomes are generally favorable with prompt diagnosis and source control, but delays can lead to substantial morbidity [4, 5, 13, 95]. Most adults recover fully with early joint irrigation and culture-directed antimicrobials, whereas outcomes worsen with older age, a higher Charlson comorbidity index, and pre-existing joint diseases such as OA and RA [4, 13]. A recent multicenter cohort using a desirability of outcome ranking framework demonstrated that delayed surgical intervention was associated with higher 1-year mortality and treatment-failure rates [6]. This composite approach, integrating survival, relapse, reinfection, and joint recovery, reinforces expert guidance advocating early debridement as a determinant of both survival and long-term function. Case fatality rates remain low in otherwise healthy adults but can reach approximately 20% in older or comorbid populations [4]. Mortality correlates strongly with treatment delay, uncontrolled sepsis, and underlying systemic illness, emphasizing NJSA as a time-sensitive orthopedic and infectious disease emergency.

### Functional Outcomes and Long-Term Complications

Functional recovery is increasingly viewed as co-equal to microbiologic cure. Approximately 20%–50% of patients fail to regain baseline function and residual stiffness or chronic pain is common [6, 95]. OA progression appears accelerated following NJSA with knee arthroplasty rates increased up to 27-fold compared with age-matched populations [4].

Functional prognosis varies by joint. Among large joints, the shoulder is associated with the poorest functional outcomes. In 1 comparative study, only 50% of patients with shoulder NJSA returned to prior activity levels compared with 71% of individuals with knee NJSA [101]. Although the knee is more common and generally demonstrates better functional preservation than the shoulder or hip, substantial long-term morbidity persists [4].

Physical therapy is critical in preserving joint function once acute infection is controlled with early mobilization being essential to prevent stiffness, arthrofibrosis, and long-term disability [10]. Rehabilitation should begin with pain-adapted passive range-of-motion exercises progressing to active mobilization as tolerated with muscle strengthening introduced later. Although comparative data on specific rehabilitation strategies in NJSA are lacking, surgical series emphasize the importance of supervised physical therapy as early as postoperative day 1 to minimize stiffness and long-term functional impairment [10, 102].

## MULTIDISCIPLINARY MANAGEMENT

Effective management of NJSA requires coordinated multidisciplinary care, beginning with frontline clinicians who prioritize early recognition, prompt synovial fluid sampling and blood cultures before antimicrobials, and urgently request orthopedic evaluation to expedite operative source control and provide key intraoperative findings that guide subsequent management.

Early and sustained infectious diseases consultation is associated with improved outcomes across musculoskeletal infections, including lower recurrence rates, shorter hospital stays, and more appropriate antimicrobial use [103, 104]. Where available, Orthopedic Infectious Diseases subspecialists further enhance care by addressing musculoskeletal infection nuances, facilitating communication with surgical teams, implementing standardized protocols, and ensuring longitudinal multidisciplinary follow-up [105–107]. Rheumatology specialists may primarily manage NJSA in some clinical settings but remain essential when crystalline or autoimmune disease mimics NJSA, enabling accurate diagnosis without delaying joint-saving interventions [10, 12]. For PWID, Addiction Medicine specialists, nursing, case management, and social services facilitate harm-reduction strategies, opioid agonist therapy, and individualized dismissal planning to improve treatment completion and reduce readmissions [25]. Together, these disciplines form a cohesive NJSA care framework that improves diagnostic accuracy, operative timeliness, patient-centered follow-up and long-term outcomes.

## FUTURE DIRECTIONS AND RESEARCH PRIORITIES

Despite advances in multidisciplinary care, much of the NJSA evidence base is derived from retrospective cohorts limited by heterogeneous definitions, confounding by indication, and inconsistent outcome reporting. As summarized in Table 5, unresolved questions include optimal synovial fluid thresholds, the role of molecular diagnostics and advanced imaging, timing and strategy of source control, and individualized antimicrobial duration and oral transition strategies.

**Table 5.** Priority Research Questions in Native Joint Septic Arthritis

Domain	Key Research Questions
Diagnosis	Should synovial leukocyte and differential thresholds vary by joint, pathogen, or host factors? In culture-negative NJSA, what is the clinical utility of molecular diagnostics (multiplex PCR, 16S/NGS, plasma mcfDNA), particularly for detection of atypical or fastidious pathogens? What is the role of advanced imaging (eg, FDG-PET/CT) in identifying multifocal disease (eg, endocarditis or adjacent osteomyelitis) and in evaluating persistent infection prior to discontinuation of antimicrobial therapy?
Source control	What is the impact of time to source control on treatment outcome and long-term function? In which patients is arthroscopy equivalent or superior to open arthrotomy, and what is the role of serial aspiration?
Antimicrobial Strategy	What is the optimal duration of therapy after adequate source control, and should it vary by pathogen, bacteremia, joint type, or drainage adequacy? What defines safe early oral step-down, including for multidrug resistant microorganisms?
Host factors	Should antimicrobial duration, route, or monitoring differ across host phenotypes, including persons who inject drugs, or immunocompromised patients (eg, neutropenic or oncology populations)?
Outcomes	What is the optimal definition of treatment failure and success? Can composite endpoints such as patient-reported outcomes or DOOR frameworks better capture meaningful recovery?

Abbreviations: 16S, 16S ribosomal RNA gene sequencing; DOOR, desirability of outcome ranking; FDG-PET/CT, fluorodeoxyglucose positron emission tomography/computed tomography; mcfDNA, microbial cell-free DNA; NGS, next-generation sequencing; NJSA, native joint septic arthritis; PCR, polymerase chain reaction.

Future progress will require large prospective multicenter studies and where feasible, pragmatic randomized trials to clarify optimal diagnostic and therapeutic strategies. Careful attention to host heterogeneity and joint-specific biology will be essential to refining clinical decision-making in NJSA.

## CONCLUSION

In summary, NJSA is a time-critical infection in which outcomes depend on early suspicion, prompt diagnosis, rapid source control, and individualized antimicrobial therapy delivered through coordinated multidisciplinary care. Although diagnostic and management strategies have evolved, the clinical utility of modern diagnostics remains incompletely defined and variability persists in treatment approaches and outcome reporting. High-quality prospective studies are needed to establish consistent evidence-based standards and improve patient outcomes.

## Supplementary Material

ciag473_Supplementary_Data

## Supplementary Data

Supplementary materials are available at *Clinical Infectious Diseases* online. Consisting of data provided by the authors to benefit the reader, the posted materials are not copyedited and are the sole responsibility of the authors, so questions or comments should be addressed to the corresponding author.
