# Supplementary material for: State-of-the-Art Review: Native Joint Septic Arthritis Epidemiology, Pathogenesis, Microbiology, Diagnosis, Management, and Outcomes
Source: Clin Infect Dis. 2026 Oct 28;83(4):499–513. doi: 10.1093/cid/ciag473 (PMC13715976; doi:10.1093/cid/ciag473)
Supplement: Supplementary file 1 [file ciag473_supplementary_data.docx]

**Supplemental Figure 1: MRI Imaging of Right Sacroiliac Joint NJSA**

**Supplemental Figure 1: MRI Imaging of Right Sacroiliac Joint NJSA**

**Abbreviations:** MRI, magnetic resonance imaging; SI, sacroiliac; NJSA, native joint septic arthritis; IDU, injection drug use.

Axial T2W fat saturated MRI image demonstrating right SI joint NJSA in a 27-year-old female with active IDU, back pain, and radiculopathy. There is evidence of increased SI joint effusion (long thin black arrows), ill-defined reactive bone marrow edema of the adjacent bones (stars). In addition, there is a fluid collection anterior to the SI joint (short thick white arrow) as well as ill-defined inflammation and edema of the adjacent soft tissues (short thick black arrows).
